# Editorial: The Evolving Telomeres

**DOI:** 10.3389/fgene.2016.00050

**Published:** 2016-04-06

**Authors:** Kurt W. Runge, Arthur J. Lustig

**Affiliations:** ^1^Department of Immunology, The Lerner Institute, Cleveland Clinic FoundationCleveland, OH, USA; ^2^Department of Biochemistry and Molecular Biology, The Tulane Medical School and Tulane Cancer CenterNew Orleans, LA, USA

**Keywords:** molecular and experimental evolution, telomere-binding proteins, telomerase, RAP1 interacting protein 1, long nuclear RNA, yeast, Arabidopsis, TRFL proteins

The study of the evolution of the end of chromosomes, or telomeres, has moved from the abstract to molecular observations and mechanistic possibilities. Although successful end-replication and end-protection are the primary driving forces acting at all telomeres (de Lange, 2009), the studies presented in this issue reveal apparent similarities, surprising differences, and new functions for telomere binding proteins (TeloBPs). These advances in molecular genetics of both common and more diverse organisms should lead to specific hypotheses for the roles of these proteins both at telomeres and throughout the genome and toward a broader view of how evolution solves different problems that occur in biology. The next step will be the experimental testing of evolutionary hypotheses.

As a reflection of the molecular advances, we framed the series “The Evolving Telomeres”. We have covered information from multiple systems that use a variety of mechanisms. These include studies in Neal Lue's lab regarding the analysis of work in yeasts belonging to Saccharomycotina involving the co-evolution of single-stranded and double-stranded sequence TeloBPs as a function of telomeric sequence (Steinberg-Neifach and Lue). They find that proteins accommodate the differing sequence through duplication and divergence of functional proteins, combinatorial site recognition, and greater protein flexibility. David Shore's laboratory reviewed the apparent differences and similarities in the Rif1 protein (Mattarocci et al.) in yeasts and humans. Rif1 was first defined in budding yeast as a negative regulator of telomere size that counteracted the activation effects of Tel1 (ATM) binding to short telomeres (Hector et al., 2007; Sabourin et al., 2007). The multi-functional Rif1, on the other hand, is delivered to the terminus in greater amounts in longer telomeres that have a greater abundance of the major yeast TeloBP, Rap1, thereby displacing Tel1 (Chang et al., 2007; Hirano et al., 2009; Martina et al., 2012). These activities form a feedback mechanism that protects the telomere against non-productive repair such as the formation of end-to-end fusions. This dynamic homeostasis acts in a cap-like function, termed the anti-checkpoint (Ribeyre and Shore, 2012). Feedback mechanisms seem to be ubiquitous among telomeres.

One major issue is the source of the many discontinuities in the evolution in plant, fungal, and mammalian telomeres. Two studies probed some of the unique characteristics of plants. Dorothy Shippen's laboratory (Nelson and Shippen) studied the participation of long nuclear RNAs in plant telomere regulation. Among these is the telomerase RNA and an entire group of related RNAs, many of which act on telomerase, even as a negative regulator. These RNAs are absent from metazoans, illustrating how the metaphyta have likely adapted the system of RNA-based regulation to telomeres. This finding may reflect the high predominance of RNA-based defense mechanisms in plants, especially against transposons present in most of the genome (Shabalina and Koonin, 2008). Karel Riha's laboratory contributed an experimental study of another example of differing solutions to end-protection (Fulcher and Riha). One issue in Arabidopsis and many other plants has been the lack of TRF-like (TRFL) factors that are so common in vertebrate cells. The major telomere binding proteins in vertebrates is TRF1, and often, TRF2. These proteins form the backbone of the shelterin complex, involved in both end-replication and protection (Karlseder et al., 2003; Wu and de Lange, 2008). The strangest observation is that TRFL are present and located at telomeres, but serve no obvious function. To rule out the possibility of functional redundancy, the authors' produced genetic knockouts of the possible functional TRF-like proteins with no effect on telomeres or growth. This result is in sharp contrast to the effects of TRF1 and TRF2 loss in vertebrates. Their data all but eliminate the chance for the presence that a homolog to the vertebrate telomere repeat factor (TRF1) that is important at Arabidopsis telomeres (Shakirov et al., 2008). Rather, a simple algal-related protein performs many of the TRF1 functions in Arabidopsis (Mozgova et al., 2008), leading to speculation on the odd rapid evolution of TeloBPs. Plants appear to have adapted telomeres to physiological requirements since the divergence of the original common ancestor that gave rise to metazoans.

Some components of telomeres are conserved such as the Mre11/Rad50/NBS complex and the Cdt1/Stn1Ten1 complex that assist in end protection. However, many others rapidly change with differing physiological and selective forces that maintain genome stability and cell survival. Art Lustig presented a hypothesis that evolution could cause rapid changes as a consequence of formation and divergence of paralogs (Lustig). The hypothesis argues that rapid evolution is driven by the requirement for genomic stability and, in some cases, by telomere stress response that increases the rate of paralogy and divergence. In fact, this result helps to explain the TeloBP divergence among fungal, invertebrates, vertebrate and plant species that have been investigated.

Evolution has provided multiple solutions to the end-replication problem of linear chromosomes besides telomerase and even telomeres. Some bacteriophages replicate the end by circularization or recombination (Lopes et al., 2010). Both adenovirus and the bacterium that causes Lyme disease, *Borrelia burgdorferi*, have chromosome ends capped by covalently bound proteins (Chaconas, 2005), and *Drosophila* and other dipterans have transposons at their chromosome termini (Villasante et al., 2008). The role of non-LTR retro-transposition in the evolution of telomerase has been controversial.

Indeed, in analyzing the origin of telomerase, (de Lange) proposes a theoretical scheme for type II introns, coupled with the formation of primitive t-loops, to evolve into telomerase, independent of non-LTR retro-transpositions (Lambowitz and Belfort, 2015). Nevertheless, the review by Servant and Deininger focuses on the use in extant organisms of non-LTR retro-transposition in telomerase-positive cells, providing an example of a mechanism that persists and even co-exists with telomerase through evolution. The bottom line of these studies is the diversity of telomeric processes. This variety could be put into a broader context by a more extensive study of diverse organisms.

A major future goal, at least for microbes, is to test hypotheses regarding telomere evolution. These experiments use techniques for growth of cells at a constant density. One of these instruments used for these experiments is the turbidostat (Gresham and Dunham, 2014; Matteau et al., 2015; Takahashi et al., 2015) that can differentiate between the altered molecular changes that arise during the evolution of cells. Another exciting aspect of this work is that these experiments represent real-time (albeit manipulated) evolution. The artificial evolutionary approach is having signs of success in yeast and microbes under different conditions, such as oxidative stress (Raso et al., 2012) and these successes will undoubtedly continue.

## Author contributions

KWR was responsible for background and analysis of contributions. AJL was responsible for the structure and comments in the editorial.

## Funding

Funding for theoretical studies was provided by NIH 5R01 GM069943, the Louisiana Cancer Research Consortium and pilot funds from Tulane University (to AJL). Funding was additionally provided by NSF 1516220 and NIA RO1 AG051601 (to KWR).

### Conflict of interest statement

The authors declare that the research was conducted in the absence of any commercial or financial relationships that could be construed as a potential conflict of interest.
